# Supplementary figures and images for: Evaluation of the clinical utility of the PromarkerD in-vitro test in predicting diabetic kidney disease and rapid renal decline through a conjoint analysis
Source: PLoS One. 2022 Aug 1;17(8):e0271740. doi: 10.1371/journal.pone.0271740 (PMC9342737; doi:10.1371/journal.pone.0271740)

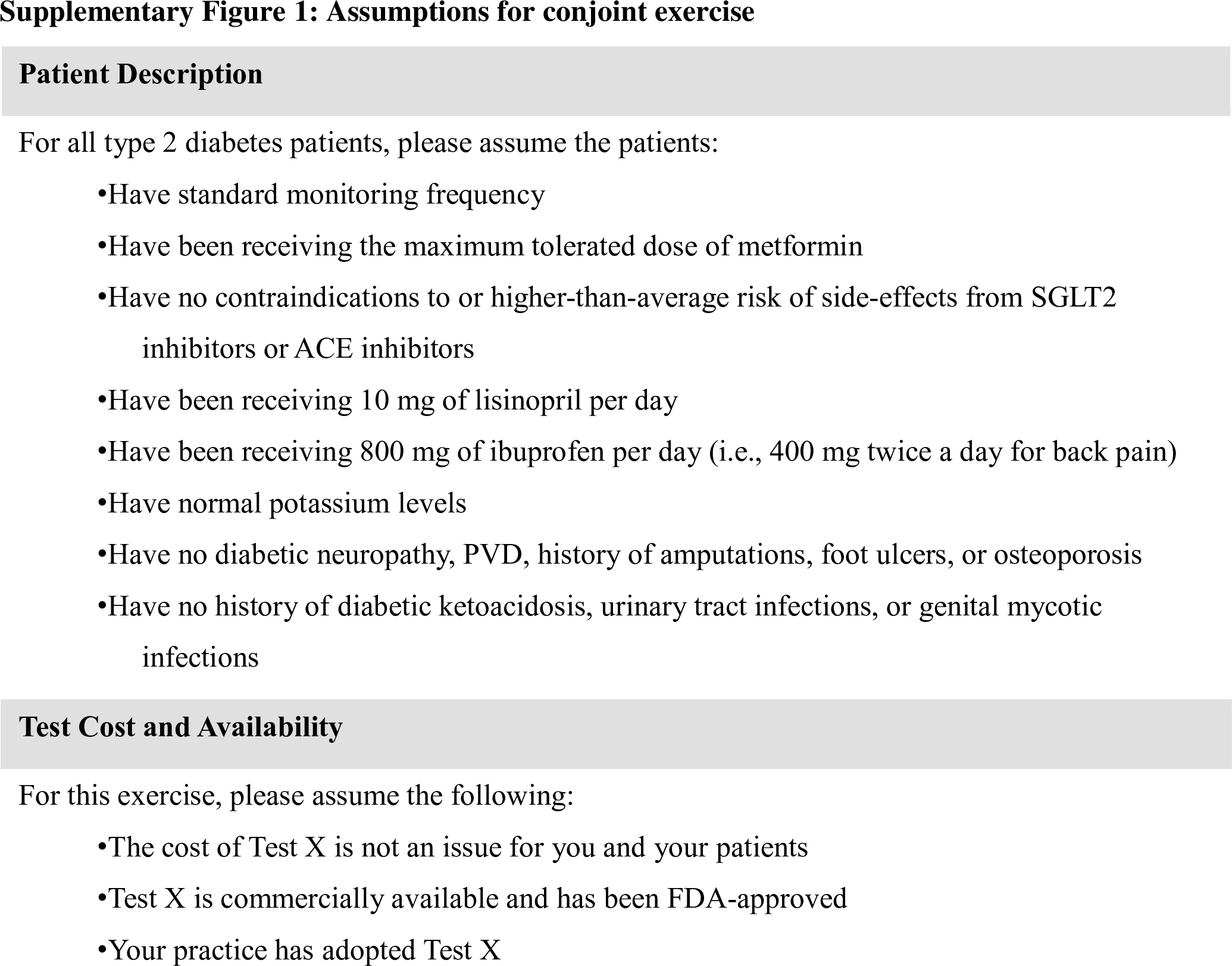

Supplement: S1 Fig — (TIF) [file pone.0271740.s001.tif]

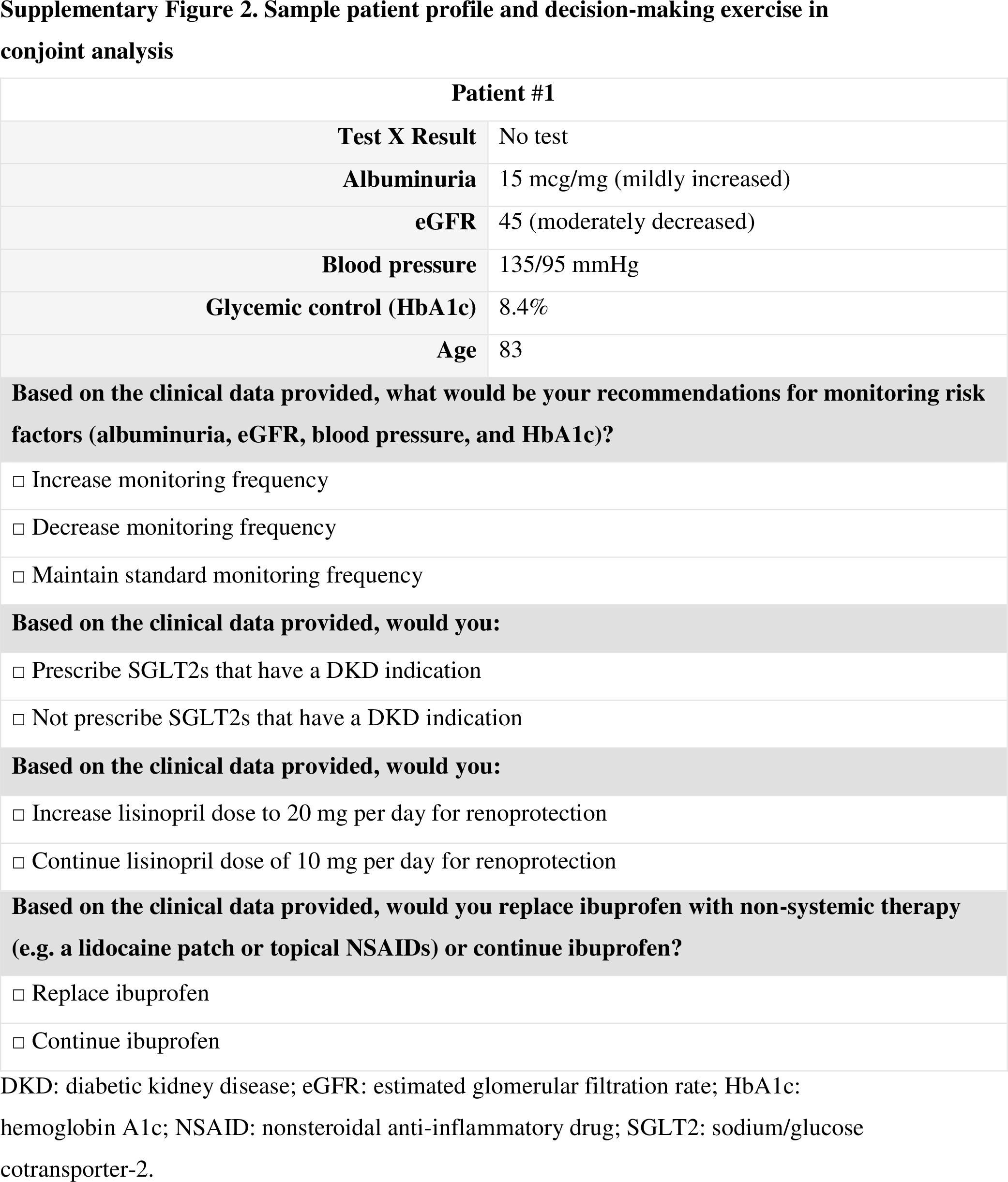

Supplement: S2 Fig — (TIF) [file pone.0271740.s002.tif]

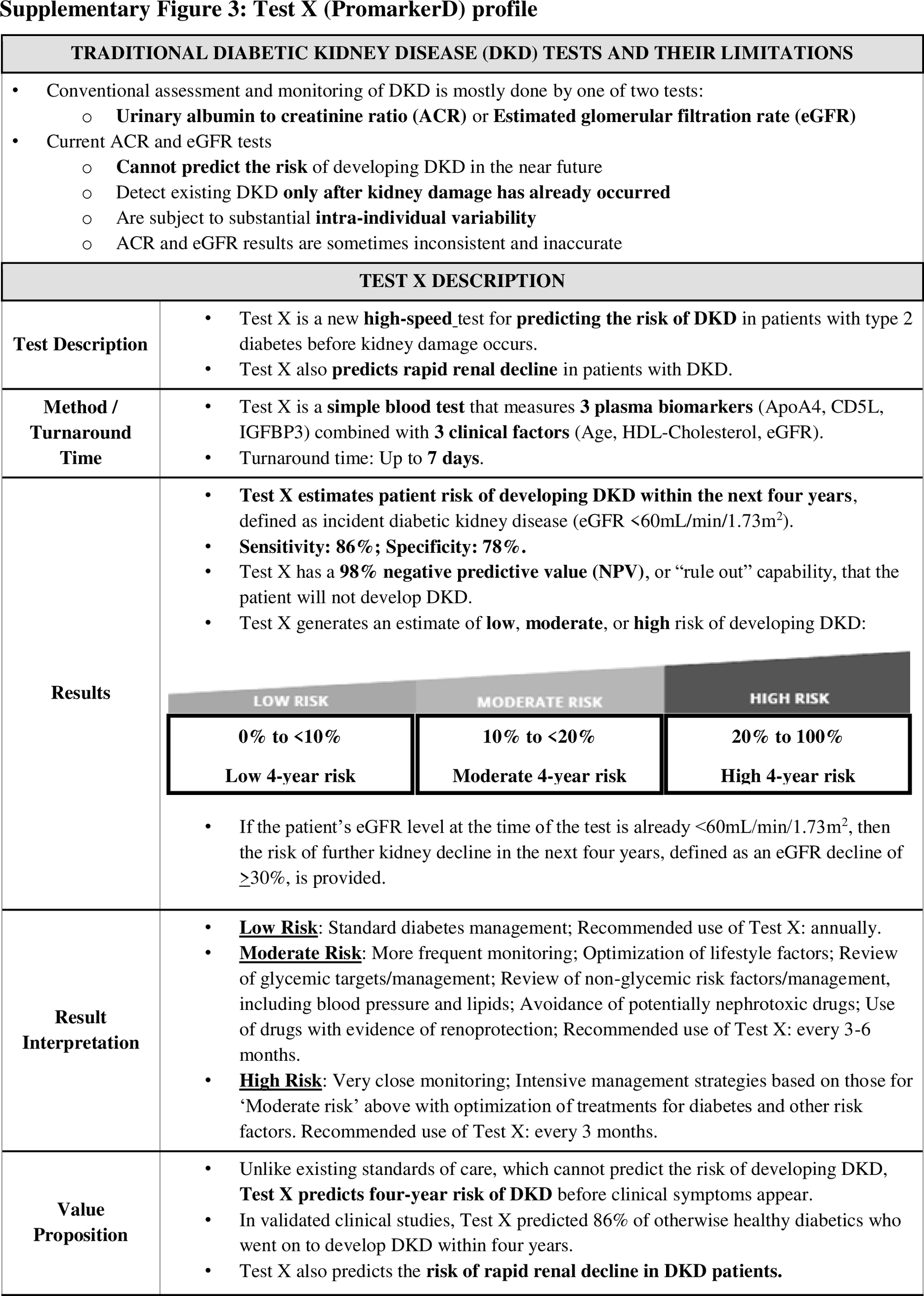

Supplement: S3 Fig — (TIF) [file pone.0271740.s003.tif]
