# Supplementary material for: Evaluation of the clinical utility of the PromarkerD in-vitro test in predicting diabetic kidney disease and rapid renal decline through a conjoint analysis
Source: PLoS One. 2022 Aug 1;17(8):e0271740. doi: 10.1371/journal.pone.0271740 (PMC9342737; doi:10.1371/journal.pone.0271740)
Supplement: S1 Table — (DOCX) [file pone.0271740.s004.docx]

**Supplementary Table 1: Association of clinical variables and PromarkerD results with increasing monitoring from standard monitoring frequency (n=400)**

^
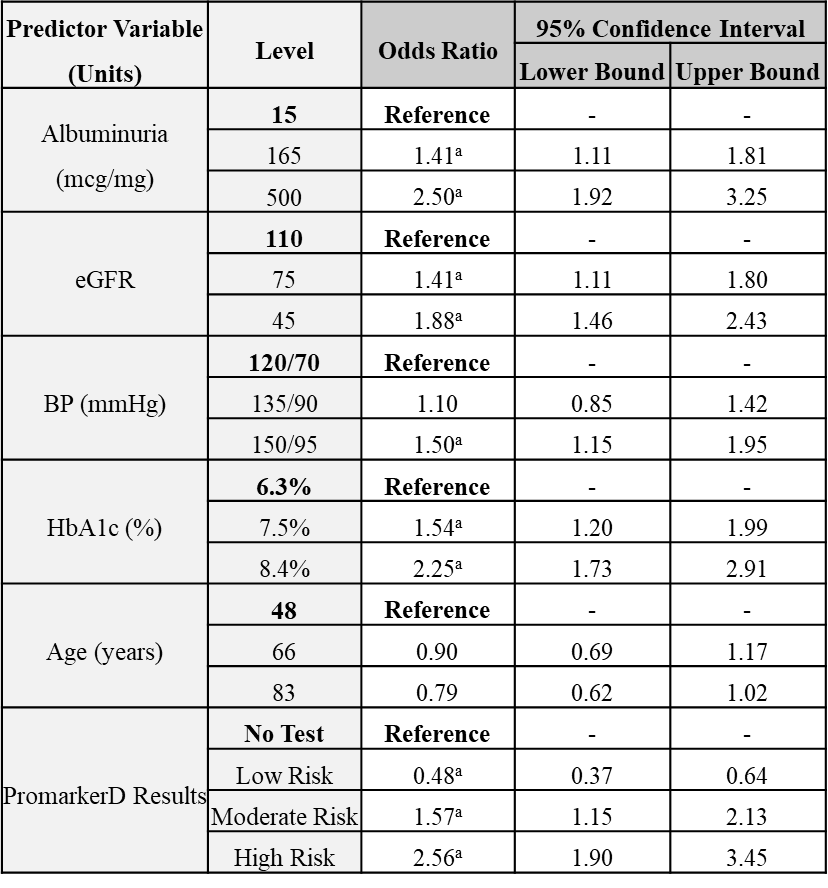
a^Significant at α<0.05 (i.e., the confidence interval does not include 1).

BP: blood pressure; eGFR: estimated glomerular filtration rate; HbA1c: hemoglobin A1c.
