# Supplementary material for: Evaluation of the clinical utility of the PromarkerD in-vitro test in predicting diabetic kidney disease and rapid renal decline through a conjoint analysis
Source: PLoS One. 2022 Aug 1;17(8):e0271740. doi: 10.1371/journal.pone.0271740 (PMC9342737; doi:10.1371/journal.pone.0271740)
Supplement: Attachment — Submitted filename: Response to Reviewers.docx [file pone.0271740.s009.docx]

PLOS ONE

Via Electronic Submission

March 24, 2022

Dear Dr. Royle,

We appreciate your consideration of our manuscript, “Evaluation of the clinical utility of the PromarkerD in-vitro test in predicting diabetic kidney disease and rapid renal decline through a conjoint analysis,” for publication by PLOS ONE, and we thank you and the reviewers for your comments and recommendations.

The authors have provided responses to the reviewers’ comments below in blue and have indicated how we have addressed these comments in the revised manuscript, where applicable. We have also uploaded the revised manuscript with track changes, as well as another revised version with changes accepted.

At the end of this letter we include a list of supplementary publications not included in the original manuscript. Several of these studies, which have been published in PLOS ONE and other respected journals, include examples of the use of conjoint/discrete choice methodology in medical research and can serve as a reference and further context for our manuscript and the approach described therein.

With kind regards on behalf of all authors,

Lauren Fusfeld

Boston Healthcare Associates (a Veranex Company)

***Review Comments to the Authors*** *Please use the space provided to explain your answers to the questions above. You may also include additional comments for the author, including concerns about dual publication, research ethics, or publication ethics. (Please upload your review as an attachment if it exceeds 20,000 characters)*

*Reviewer #1: This study evaluated the impact to physicians of PromarkerD, a biomarker-based blood test predicting the risk of diabetic kidney disease and rapid renal decline. 400 physicians (203 PCPs and 197 endocrinologists) were surveyed, and the investigators found through conjoint analyses the relative importance of ProMarkerD in making treatment decisions (starting SGLT2i, increasing ACEi dose, or discontinuing nephrotoxins). The analyses are straight forward and easy to understand. The limitations are well-delineated.

There is, however, a major limitation. The test description in Supplementary Figure 3 only describe the performance characteristics of PromarkerD for developing DKD (e.g., new incident DKD).*

*However, Level 2 and 3 attributes for albuminuria (165 and 500 mcg/mg) and level 3 attribute for eGFR (45 ml/min) would indicate prevalent DKD, and there the performance that matters is risk of progression. That is not well described in Supplementary Figure 3. (there are no statistics shown with the risk for 30% decline in eGFR). Thus, it appears that the analyses and surveys were largely applied to hypothetical patients outside the intended use of the test?*

**Author response:** Thank you for your thoughtful review and comments.

The test is intended to be used both with patients who are at risk of DKD and patients with DKD who are at risk of further renal decline. At the time the survey was in the field, we did not have the performance characteristics of PromarkerD specifically for disease progression with patients who already have DKD. Since we wanted to present validated accuracy data as opposed to hypothetical accuracy data, we provided the sensitivity, specificity, and NPV numbers associated with the risk of developing DKD, while noting in Supplementary Figure 3, *“If the patient’s eGFR level at the time of the test is already <60mL/min/1.73m2, then the risk of further kidney decline in the next four years, defined as an eGFR decline of >30%, is provided.”* Prior to launching the survey, two diabetic kidney disease clinical experts (MS and AT) were instrumental in reviewing the profile of PromarkerD for clarity and completeness for the intended audience of endocrinologists and primary care physicians. Later, during video calls with Boston Healthcare, five physicians pilot tested the survey without expressing confusion about the profile of PromarkerD or concern that the data provided in the profile impeded their ability to provide an educated assessment of their medical decisions for each hypothetical patient. In addition, none of the respondents who completed the survey indicated in the open-ended text questions that the test description was inadequate.

Since the time the survey was fielded, additional performance data have become available. The sensitivity (87%), specificity (83%), and negative predictive value (97%) associated with a combined incident DKD or eGFR30 result are similar to the values for incident DKD alone: sensitivity (86%), specificity (78%), and negative predictive value (98%). We cannot change Supplementary Figure 3 because this figure contains the information the physician respondents actually viewed, but we can add the additional accuracy data. These additional values are now on page 10, lines 179-184.

*I would suggest separate analyses that stratify those that did not yet have DKD vs. those that already have DKD.*

**Author response:** While it could be interesting to design and field additional conjoint studies that test the impact of PromarkerD and other attributes with patients at risk of DKD separately from patients who already have DKD, we cannot conduct this analysis post-hoc with the data available. As noted on page 8, lines 137-144, the conjoint design of 42 unique patient profiles was originally created because the design had a D-efficiency of 0.97 (indicating that the subset was representative of the full factorial design) based on all the attributes and levels (including eGFR) and because it was possible to minimize standard errors with the given sample size of 400 respondents.

Reviewer #2: In the present manuscript, authors aimed to evaluate how PromarkerD test would impact physicians’ prescribing and monitoring decisions for type 2 diabetes mellitus patients. Although identification of novel non-invasive biomarkers represents a challenge in the field of diabetic kidney disease, however the study presents several limitations.
Did authors evaluated in a cohort of real patients up if decisions made based on PromarkerD reduced the progression of renal damage in the follow in those patients indicated at high risk and had no impact on renal decline in patient estimated at low risk?

**Author response:** Thank you for your thoughtful review and comments.

We agree that a clinical study like the one you propose using real patients could be helpful in the future. On page 19, lines 366-368, the manuscript acknowledges, “the study did not assess type 2 diabetes mellitus patient outcomes following physician decisions. Additional research will need to be conducted to assess these outcomes.” This research could take the form of a clinical trial such as the one you had described.

The downside of using a clinical study for the initial research, however, is that such a study requires significant resources and costs, particularly during this COVID era. The clinical utility study described in this manuscript was intended to assess whether physicians will use the test in their decision making; the study was not intended to be an assessment the impact of the test on patient outcomes using data from a chart review.

A conjoint approach is an ISPOR-approved way to understand physicians’ motivation; this validated methodology, using clinical vignettes, was developed to understand implicit decision making and to mimic the real world, as noted on page 18, lines 363-364, (Bridges 2011). This conjoint/discrete choice approach has been proven to be a pragmatic, cost-effective, and time-efficient methodology for firms developing new medical technology. PLOS ONE has published these types of studies in a variety of disease areas (Boone 2013, de Vries 2015, Harrison 2019, Hazlewood 2020; Kromer 2015, and Ride 2016), as have other respected journals (Janssen 2018, Szeinback 2008), noted on page 19, lines 364-366.

An important point is the prediction of rapid renal decline in patients with diabetic kidney disease, since DKD includes different kinds of renal damages with different pathogenesis mechanisms and different treatments. In most of the cases only renal biopsy can estimate the real cause of DKD and support treatment decisions. Thus described cases could not really represent real-life.

**Author response:** The literature indicates that physicians are making treatment decisions based on KDIGO and other guidelines and that there is a need for a test like this (Datar 2021). Very few patients with suspected DKD have kidney biopsies in real-life practice of medicine (Looker 2018). In the personal experience of both diabetic kidney disease clinical experts (MS and AT), kidney biopsies are only considered by nephrologists when etiology other than DKD is suspected.

These two diabetic kidney disease clinical experts (MS and AT) were involved in developing the attributes and levels to ensure that the patient profiles were realistic and sufficiently informative for physicians to estimate their medical decisions for each hypothetical patient profile. Five physicians pilot tested the survey without indicating that the data provided in the patient profiles were insufficient for them to provide an informed medical decision. In addition, none of the respondents who completed the survey indicated in the open-ended text questions that the profiles were inadequate.

It is not clear to me how interpret the PromarkerD results and how levels were assigned.

**Author response:** PromarkerD measures 3 plasma biomarkers (ApoA4, CD5L, IGFBP3) combined with 3 clinical factors (age, HDL-cholesterol, eGFR). These data are submitted to the PromarkerD Hub, a software tool that contains an algorithm that generates a low-, moderate-, or high-risk result and provides an interpretation of the risk score. This information regarding how PromarkerD assigns levels can be seen on page 6, lines 86-91. Further details regarding the test are outside the scope of the current manuscript but have been published elsewhere (Bringans 2017; Peters 2017; Peters 2019).

Supplementary Figure 3 provides an explanation of how to interpret the test results. For example, a low-risk result indicates the 4-year risk of developing DKD (or further renal decline for patients with DKD) ranges from 0% to <10%. Similarly, a moderate-risk result indicates the 4-year risk of developing DKD (or further renal decline for patients with DKD) ranges from 10% to <20%, while a high-risk result from PromarkerD indicates the 4-year risk of developing DKD (or further renal decline for patients with DKD) ranges from 20% to 100%.

They should include a range for each attribute to assign low risk or moderate risk…

**Author response:** As noted on pages 10, lines 190-192, although the original text describing the attribute levels included ranges for each level, pilot interviews indicated ranges were too difficult to evaluate, so we replaced the ranges with point estimates.

Moreover, why sex was not considered among attribute?

**Author response:** We conducted a targeted literature review to identify the patient attributes with the largest impact on physician decision-making for type 2 diabetes mellitus patients; we then narrowed the list of attributes based on discussions with two diabetic kidney disease clinical experts (MS and AT). Including too many attributes would unduly complicate the conjoint choice exercise and would require each physician to review a burdensome number of profiles and/or necessitate an increase in the sample size.

Sex, in particular, did not qualify for the list of attributes submitted to the clinical experts for review. The impact of sex in the risk of DKD and the risk of DKD progression is unclear. Maric-Bilkan (2020) notes, *“Particularly, women with diabetes have higher mortality rates for diabetes-related deaths, and higher prevalence of diabetic kidney disease risk factors such as hypertension, hyperglycemia, obesity, and dyslipidemia.* ***However, the evidence for the impact of sex on diabetic kidney disease prevalence and disease progression is limited and inconsistent.*** *Although most studies agree that the protective effect of the female sex against the development of kidney disease is diminished in the setting of diabetes, the reasons for this observation are unclear.* ***Whether or not sex differences exist in the risk of diabetic kidney disease is also unclear, with studies reporting either higher risk in men, women, or no sex differences.”***

Number of diabetic kidney disease clinical experts for treatment decision is limited. Where they nephrologist or endocrinologist?

**Author response:** Both clinical experts involved in developing the primary research materials for this study are endocrinologists. We also spoke with five physicians (three endocrinologists and two primary care physicians) in the pilot phase of this study. As noted in Table 2 on pages 12 and 13, 400 additional physicians (197 endocrinologists and 203 primary care physicians) are included in the analysis.

PromarkerD can help primary care physicians and endocrinologists, the target clinicians in this study, decide whether to refer patients to nephrologists.

An important limitation of the survey is also the lack of informations regarding data collection methods, test result interpretations, test validation and costs since a correct evaluation on the decision impact of PromarkerD results versus no test should also consider time and costs needed to obtain data.

**Author response:** For data collection, in a real-life clinical setting, the process is as follows:

Clinical Process

1. A patient with type 2 diabetes mellitus would be seen regularly by the managing clinician (most commonly a general practitioner) for general monitoring and annual standard of care tests, including eGFR and uACR, to assess kidney function.
2. The managing clinician would request PromarkerD for patients with a recent history of eGFR and HDL-cholesterol results.
3. Patients would be referred for a blood draw.
4. Blood samples would be sent to accredited laboratories where the PromarkerD kit would be used to measure each protein biomarker in the sample via enzyme-linked immunoassay (ELISA).
5. Test results would be interpreted and uploaded into the PromarkerD hub to produce a risk score.
6. The PromarkerD risk score would be provided to managing clinicians who would then relay that information to patients.
7. The test risk score would inform further patient care such as monitoring frequency, lifestyle modification, addition of other medications, initiation of more aggressive treatment measures, and patient education on risk factors.

Supplementary Figure 3 presents test result interpretation guidelines for physician, which are based on well-established KDIGO guidelines.

We agree that in practice clinicians consider many factors, including time and cost, when making patient management decisions. However, we would like to emphasize that this study was intended to be a clinical survey involving hypothetical patients to assess potential use of PromarkerD and to determine where physicians saw clinical value. Assumptions for the choice exercise in survey are provided in Supplementary Figure 1, including patient description, test cost (“The cost of Test X is not an issue for you and your patients”), and test availability. Further details on test validation are outside the scope of the current manuscript but have been published elsewhere (Bringans 2017; Peters 2017; Peters 2019). PromarkerD’s price in the United States has not been determined; a full cost-effectiveness model is currently underway.

Additional Publications Referenced in this Letter but Not Included in the Manuscript

*Conjoint Studies in Medical Research*

- Boone D, Mallett S, Zhu S, et al. Patients’ & healthcare professionals’ values regarding true- & false-positive diagnosis when colorectal cancer screening by CT colonography: discrete choice experiment. PLOS ONE. 2013;8(12):e80767.
- de Vries ST, de Vries FM, Dekker T, et al. The role of patients’ age on their preferences for choosing additional blood pressure-lowering drugs: a discrete choice experiment in patients with diabetes. PLOS ONE. 2015;10(10):e0139755.
- Harrison M, Spooner L, Bansback N, et al. Preventing rheumatoid arthritis: Preferences for and predicted uptake of preventive treatments among high risk individuals. PLOS ONE. 2019;14(4):e0216075.
- Hazlewood GS, Pokharel G, Deardon R, et al. Patient preferences for maintenance therapy in Crohn’s disease: A discrete-choice experiment. PLOS ONE. 2020;15(1):e0227635.
- Kromer C, Schaarschmidt ML, Schmieder A, Herr R, Goerdt S, Peitsch WK. Patient preferences for treatment of psoriasis with biologicals: a discrete choice experiment. PLOS ONE. 2015;10(6):e0129120.
- Ride J, Lancsar E. Women’s preferences for treatment of perinatal depression and anxiety: a discrete choice experiment. PLOS ONE. 2016;11(6):e0156629.

*Study Demonstrating Need for PromarkerD*

- Datar M, Ramakrishnan S, Montgomery E, Coca SG, Vassalotti JA, Goss T. A qualitative study documenting unmet needs in the management of diabetic kidney disease (DKD) in the primary care setting. BMC Public Health. 2021;21(1):930.

*Use of Kidney Biopsies*

- Looker HC, Mauer M, Nelson RG. Role of kidney biopsies for biomarker discovery in diabetic kidney disease. Adv Chronic Kidney Dis. 2018;25(2):192-201.

*PromarkerD Validity*

- Bringans SD, Ito J, Stoll T, et al. Comprehensive mass spectrometry based biomarker discovery and validation platform as applied to diabetic kidney disease. EuPA Open Proteom. 2017;14:1-10.

*Sex Differences in Kidney Disease*

- Maric-Bilkan C. Sex differences in diabetic kidney disease. Mayo Clin Proc. 2020;95(3):587-599.
