# Supplementary material for: Evaluation of the clinical utility of the PromarkerD in-vitro test in predicting diabetic kidney disease and rapid renal decline through a conjoint analysis
Source: PLoS One. 2022 Aug 1;17(8):e0271740. doi: 10.1371/journal.pone.0271740 (PMC9342737; doi:10.1371/journal.pone.0271740)
Supplement: Attachment — Submitted filename: Response to Reviewers.docx [file pone.0271740.s010.docx]

PLOS ONE

Via Electronic Submission

June 30, 2022

Dear Dr. McGrowder,

We appreciate your consideration of our manuscript, “Evaluation of the clinical utility of the PromarkerD in-vitro test in predicting diabetic kidney disease and rapid renal decline through a conjoint analysis,” for publication by PLOS ONE, and we thank you and the reviewers for your comments and recommendations.

The authors have provided responses to the new reviewers’ comments below in blue and have indicated how we have addressed these comments in the revised manuscript, where applicable. We have also uploaded the revised manuscript with track changes, as well as another revised version with changes accepted.

To address the reviewers’ comments, we have added the following reference:

Peters K, Bringans S, Davis W, Lipscombe R, Davis T. A comparison of PromarkerD to standard-of-care tests for predicting renal decline in type 2 diabetes. JASN Conference: Kidney Week 2021 San Diego, CA United States 32. 2021;260.

As we were reviewing the references, we realized that to maintain consistency a software reference was better suited for the main manuscript text (page 11, line 201). Consequently, we have removed the following from the reference list:

Analysis C, Software S. MBC v1.1. 2016.

Also, given that we first submitted the manuscript a year ago, we have replaced two references with more recent publications. The new references are the following:

CDC. Type 2 diabetes [Internet]. 2021 Dec 15 [cited 2022 June 28]; Available from: https://www.cdc.gov/diabetes/basics/type2.html#:~:text=More%20than%2037%20million%20Americans,adults%20are%20also%20developing%20it

U.S. Census Bureau. [Internet]. U.S. and World Population Clock. 2022 [cited 2022 Jun 28]. Available from: https://www.census.gov/popclock/data_tables.php?component=growth

With kind regards on behalf of all authors,

Lauren Fusfeld

Boston Healthcare Associates (a Veranex Company)

***Review Comments to the Authors***

*[The email from PLOS ONE on 5/19/22 starts with comments from Reviewer 3.]*

Reviewer #3: In this manuscript, the authors assessed the impact of PromarkerD in clinical decision-making by conjoint analysis in diabetes mellitus patients at risk of worsening renal failure. The reviewer thinks the methodology is novel and the conclusion is well supported by the results. The authors’ response to the previous reviewers looks appropriate.

The reviewers believe that this study is of clinical importance, but would like to identify some issues that have not been raised by previous reviewers.

The authors generated 42 hypothetical patient profiles for the conjoint analysis. Although the authors showed the attributes and levels of the generated patients in Table 1, the reviewer would suggest showing the summarized data of the actual parameters of the hypothesized patients (e.g. the median age with the 95% C.I.).

**Author response:** Thank you for your thoughtful review and suggestions.

Sawtooth Software’s CVA module was used to generate a design that is orthogonal (meaning that each pair of levels across different attributes appears about an equal number of times) and balanced (meaning within each attribute, each level appears about an equal number of times). The table below shows the number of times each level of each attribute appears in the data set of this study. Based on the attribute level counts (i.e., the number of times the attribute level appears in a profile) in this table, the different age levels are almost equally represented in the conjoint design.

| **Attribute Level Counts** | | | | | | |
| --- | --- | --- | --- | --- | --- | --- |
|  | **Attributes** | | | | | |
| ***Attribute Levels*** | **Att 1 - Albuminuria (ACR) (mg/g)** | **Att 2 - eGFR** | **Att 3 - Blood Pressure** | **Att 4 - HbA1c** | **Att 5 - Age** | **Att 6 - Test result** |
| ***1*** | 993 | 1063 | 1068 | 990 | 1066 | 835 |
| ***2*** | 1143 | 992 | 1064 | 1065 | 1067 | 762 |
| ***3*** | 1064 | 1145 | 1068 | 1145 | 1067 | 768 |
| ***4*** | NA | NA | NA | NA | NA | 835 |

The full data set for the conjoint analysis has been provided as part of the submission, and the manuscript text on page 8, line 143 notes the orthogonality. We believe that describing the design in this manner is more appropriate for this type of analysis than providing median values.

PromarkerD takes age and eGFR into account, and these two items overlap among the six attributes. Is this not a confounding factor in performing a conjoint analysis? That is, would clinicians who focus on Age and eGFR tend to focus on PromarkerD?

The goal of the conjoint exercise was to understand physicians’ decision-making in an exercise designed to mimic real-world situations in which doctors have access to PromarkerD test results *and* other patient characteristics, including age and eGFR. While it is possible that clinicians who focus on age and eGFR might also focus on PromarkerD (which incorporates these factors), it is also possible that clinicians could disregard the results of PromarkerD and focus solely on currently available patient attributes. The conjoint analysis answers the question, “Are age and eGFR individually more valuable than PromarkerD, which includes them as part of a composite score?” The data indicate that the importance of PromarkerD is actually much higher than the importance of age and eGFR individually.

In addition, while we were designing the conjoint exercise, we confirmed that every level of a given attribute is realistically able to occur with every level of other attributes. Hence, no prohibitions exist between the PromarkerD results and the age levels presented (i.e., the low, moderate, and high PromarkerD results could occur with any of the ages in the conjoint analysis); likewise, no prohibitions exist between the PromarkerD results and the eGFR results presented.

The “Descriptive Findings” contents are difficult to grasp on first reading. Among the supplemental figures (S4-6), the reviewer would suggest that S5 and S6 be combined into one figure and upgraded to the main figure to help readers understand the contents.

Thank you for your suggestion. Because the original Supplemental figures S4-6 provide background information about the current state of care (tests conducted in type 2 diabetes patients and monitoring frequency in patients at low and increased risk for diabetic kidney disease) rather than information about the conjoint exercise itself, we had originally placed the figures in the Supplemental section. However, we do want to make the findings as easy to understand as possible, so we have moved them into the main document at your recommendation. Supplemental figure S4 is now Figure 2 and is mentioned on page 13, lines 248-249. We also appreciate that it may be useful to compare the results of S5 and S6, so we have combined the figures into a single figure in the main text (mentioned on Figure 3, page 14, line 250) as you have suggested.

Reviewer #4: Fusfeld et al. evaluated the clinical utility of the PromarkerD in 400 physicians for predicting the risk of diabetic kidney disease and rapid renal decline. PromarkerD can successfully predict patients who develop chronic kidney disease (CKD) from previous studies. However, in Type 2 Diabetes in the Canagliflozin Cardiovascular Assessment Study (CANVAS), PromarkerD can predict incident CKD but had limited utility for predicting eGFR decline ≥30% (J Clin Med. 2020 Oct 6;9(10):3212. doi: 10.3390/jcm9103212.). Therefore, it is not well documented for the prediction or rapidly renal function decline. The interpretation of this part should be cautious.

**Author response:** Thank you for your methodical review and recommendations.

We acknowledge that the performance of the eGFR decline ≥30% component of PromarkerD was poorer in CANVAS than in previous studies, but a discussion of this was beyond the scope of the present study. Nevertheless, one possible cause of the performance difference is the variation in the distribution of PromarkerD scores with respect to cut-off values across studies. In CANVAS, the distribution of PromarkerD scores for the eGFR decline ≥30% outcome was highly skewed, with most participants (>98%) having low-risk scores and none having high-risk scores. Given this skewed distribution, there was limited power to detect even small effect sizes for PromarkerD moderate-risk scores versus low-risk scores on outcome. Nevertheless, higher PromarkerD scores were predictive of outcome, when analyzed as a continuous score from 0-100% (OR 1.13 95% CI 1.04-1.24), indicating that PromarkerD was indeed able to predict eGFR decline ≥30%, and that the risk cut-offs may need adjusting in some populations.

There are also several demographic and clinical differences between CANVAS and FDS2. PromarkerD was developed in a relatively healthy population of people with well-controlled type 2 diabetes. Subjects in CANVAS had higher glycated hemoglobin and either had a history of cardiovascular disease or were at high-risk of future cardiovascular events based on risk factors (long diabetes duration, hypertension, current smoking, albuminuria, or low serum HDL-cholesterol). In contrast, FDS2 is a community-based cohort that is representative of patients with T2D from an urban Australian population. Compared with FDS2, CANVAS participants at baseline were younger (mean age 62.7 versus 65.5 years), more were male (67.0% versus 54.3%), the median glycated hemoglobin was higher (8.0% versus 6.8%), and the median diabetes duration was longer (12.4 versus 7.0 years). In terms of baseline renal function, CANVAS participants had more renal impairment (16.5% with eGFR <60 mL/min/1.73m2) but less albuminuria (27.8% with uACR ≥30 mg/g) compared with those in FDS2 (11.6% with renal impairment and 34.2% with albuminuria).

Besides, what is the recommendation for additional treatment strategy when patients had a moderate or higher risk PromarkerD result.

Supplementary figure 3 (mentioned on page 10, line 181) describes the treatment strategy after moderate-risk results and high-risk results. The treatment strategy is based on the consensus report from the American Diabetes Association and the European Association for the Study of Diabetes (Davies MJ et al. Management of hyperglycemia in type 2 diabetes, 2018. A consensus report by the American Diabetes Association (ADA) and the European Association for the Study of Diabetes (EASD). *Diabetes Care.* 2018;(41):2669–2701.)

The treatment strategy for patients with a moderate-risk result includes the following: more frequent risk factor monitoring (every 3-6 months); optimization of lifestyle factors (nutrition, weight loss, exercise, and quitting smoking/drinking); a review of glycemic targets/management; review of non-glycemic risk factors/management, including blood pressure (e.g., ACEi, ARBs) and lipids (e.g., statins); avoidance of potentially nephrotoxic drugs (e.g., NSAIDs); and consideration of the use of drugs with evidence of renoprotection (e.g., SGLT2i, GLP1ra).

The treatment strategy for patients with a high-risk result includes the following: very close risk factor monitoring (every 1-3 months); intensive management strategies based on those for moderate risk, with optimization of treatments for diabetes and other risk factors; and possible referral to a nephrologist for maximal intervention.

A clinical implication could also be emphasized in the discussion section.

We agree the clinical impact of PromarkerD is important. We have provided commentary on the clinical impact in the original submission in the Discussion section on page 17, lines 318-321; the text in this section states, *“With accurate risk prediction, physicians would be able to administer optimal interventions and treatments at the appropriate time, understand when to prioritize the avoidance of nephrotoxic drugs, and tailor risk factor monitoring frequency to each patient.”* Also, on page 18, lines 347-355, the Discussion section notes, *“A low-risk PromarkerD result would significantly reduce the likelihood of aggressive patient management compared with no test, suggesting PromarkerD may reduce unnecessary treatments, limiting adverse effects and saving time and costs. Similarly, a PromarkerD moderate- and high-risk result would significantly increase the likelihood of renoprotective treatment changes by physicians and would support a more personalized approach to diabetic kidney disease management. The importance of using elevated PromarkerD risk results to identify patients at risk of diabetic kidney disease before renal damage occurs could increase even further in the future as new medications are developed for kidney disease in type 2 diabetes mellitus patients.”*

Reviewer #5: Another major limitation is that only 400 physicians out of approximately 8,000 physicians who were invited eventually joined the study. Please mention the bias in the Discussion section.

**Author response:** Thank you for your recommendation.

While 7,851 invitations to the survey were sent, 691 opened the email invitation. (We do not know if the unopened invitations were seen by the physicians.) We have added text on the potential for bias in the Discussion section on page 19, lines 374-381.

Reviewer #6: (No Response)

Reviewer #7: Comments
1. This study attempted to assess the impact of changes in physician practice patterns based on PromarkerD, a study that is becoming well established. However, since PromarkerD is anonymized with Test T, the usefulness of PromarkerD is not disclosed. This is hardly a conclusion that can be drawn from PromarkerD's previous research findings, and participants would have imagined a perfect test with no deficiencies.

**Author response:** Thank you for taking the time to provide comments.

To ensure that all respondents were basing their responses on the same information about PromarkerD (as opposed to other data or varying conceptions of the test), respondents were presented with a single anonymized description of the test. The test description does include the usefulness of PromarkerD, and the description does not state the test has perfect accuracy. Supplementary figure 3 (mentioned on page 10, line 182) notes, *“Unlike existing standards of care, which cannot predict the risk of developing DKD, Test X predicts four-year risk of DKD before clinical symptoms appear. In validated clinical studies, Test X predicted 86% of otherwise healthy diabetics who went on to develop DKD within four years. Test X also predicts the risk of rapid renal decline in DKD patients.”* In addition, Supplementary figure 3 notes the sensitivity is 86%, the specificity is 78%, and the negative predictive value is 98%.

2. The responses examined in this study were probably influenced more by basic knowledge, such as whether or not ibuprofen is known to have an effect on renal dysfunction, than by changes in responses due to PromarkerD (TEST X). In order to evaluate the usefulness of PromarkerD, it would be necessary to evaluate the effectiveness of the model without PromarkerD values (association between various parameters and practice patterns for the No TEST cases) and the model with added PromarkerD values for changes in practice patterns.

The model already accounts for the impact on physician medical decisions of having Test X results (low-risk, moderate-risk, or high-risk) versus not having any Test X results at all. For the decision to replace ibuprofen, for example, the 95% confidence intervals of the odds ratios for low-, moderate-, and high-risk Test X results exclude 1 (the odds ratio representing the reference level of “no test”). The implication is that the test *result* impacted the decision to replace ibuprofen, as the odds ratios summarize the impact of the test while controlling for patient attributes. Please refer to Figure 5 (mentioned on page 16, line 310), Supplementary table 1D (mentioned on page 16, line 296), and page 16, lines 295-309 of the manuscript for details on the odds ratios.

Reviewer #8: This article describes the utility of PromarkerD, a biomarker-based blood test, in clinical decision making for monitoring renal function in diabetic patients. They used conjoint analysis, a method that derives attribute importance based on respondents' selection of hypothetical outcomes using standardized virtual vignettes (p. 7). After analyzing a web-based survey of decisions of 400 physicians to treat eight from forty-two hypothetical patient profiles, they found that PromarkerD was most or second most important in the decision of treatment strategies including increasing the frequency of risk factor monitoring, prescribing SGLT2s inhibitors with a diabetic kidney disease indication, increasing the dose of lisinopril, and replacing ibuprofen with a non-nephrotoxic medication. They concluded that PromarkerD could increase adoption of renoprotective interventions in patients at high risk and lower the likelihood of aggressive treatment in those at low risk for renal decline.

This study is well designed and clearly explained. The response to reviewers is acceptable.
The reviewer has some minor comments as follows.

In the abstract, it is concluded that PromarkerD could increase adoption of renoprotective interventions in patients at high risk and lower the likelihood of aggressive treatment in those at low risk for renal decline. However, as discussed in the revision, this virtual study could not assess the patient outcomes following physician decisions. Therefore, after the conclusion, the need for further clinical studies to assess patient outcomes after applying this PromarkerD in real-world practice should be described in the abstract. This will clarify the limitations of the study design and help readers understand the current position of PromarkerD in clinical application.

**Author response:** Thank you for your careful review and suggestions.

At your recommendation, we have added to the abstract that further clinical studies are needed to assess patient outcomes after applying PromarkerD in real-world practice on page 3, lines 33-35. (Note that to adhere to the word limit of 300 words, we needed to modify some of the other text in the abstract.)

References should be added to support the statement on page 10, lines 178-184.

We agree that the following text requires a reference, *“Similar test performance characteristics for a combined group of patients at risk for either incident DKD or progression of DKD are now available: the sensitivity, specificity, and negative predictive value associated with a combined incident DKD or eGFR30 result are 87%, 83%, and 97%, respectively.”* We have added a reference on page 10, line 186.

Reviewer #9: I read with great interest the manuscript entitled " Evaluation of the clinical utility of the PromarkerD in-vitro test in predicting diabetic kidney disease and rapid renal decline through a conjoint analysis".
This study surveyed and assessed the interventional behavior of primary care physicians and nephrologists following the results of PromarkerD, a test that was approved to predict the progression of diabetic nephropathy. They found that this marker is taken into consideration as a secondary parameter after HbA1c, blood pressure levels and eGFR to act upon prescribing SGLT2i, lisinopril or avoiding ibuprofen respectively.

This is a well-written paper. The research question is interesting and can be the first step to evaluate in the future whether the decisions of physicians based on this new marker would improve the progression of CKD in patients with diabetes.

I have two comments.

1. Minor comment to be taken into consideration or not by authors and editor (Reference: <https://kdigo.org/wp-content/uploads/2022/03/KDIGO-2022-Diabetes-Management-GL_Public-Review-draft_1Mar2022.pdf>):
Regarding the nomenclature of kidney diseases, there is a tendency, adopted by the KDIGO and that will be launched soon, suggesting to avoid the term "diabetic kidney disease" because we are never sure without a biopsy that diabetes is the main cause of chronic kidney disease. So KDIGO team will call to rather use "chronic kidney disease with diabetes". The KDIGO replaced as well the term "end-stage kidney disease" by "kidney failure". However, a lot of authors worldwide are still using the terms used in this manuscript.

**Author response:** Thank you for your thorough review and suggestions.

We appreciate you calling our attention to the new nomenclature. This manuscript was originally submitted to PLOS ONE in July 2021, before the KDIGO recommendations you cite, and throughout the original survey we use the phrase “diabetic kidney disease.” To avoid confusion, we will refrain from changing the terminology in this manuscript but will keep your recommendation in mind for future research.

2. The sample of surveyed physicians was a convenience sample. The survey was sent to 7851 physicians and 405 completed it. The authors did not mention if the sample size was representative of physicians taking care of chronic kidney disease patients with diabetes. Who is the target population in this study, is it the American pool of physicians treating diabetic kidney disease? Or is it only the 7851 selected physicians? Even if it is only the 7851 physicians and we assume a confidence level of 95%, a margin of error of 4% and the fact that 50% of the physicians treat CKD patients with diabetes, the sample size should be 558 physicians. The authors need to discuss this in the limitations of the study.

We appreciate this question, as it is an important one to address. If this were a simple regression model at the clinician level, your method of calculating sample size would be accurate. However, this analysis is based on a multi-level model design involving multiple patient profiles that are nested within a single clinician. In other words, this is a patient profile level analysis rather than just a physician level analysis.

The final multi-level design was determined using dummy data, executing the regression models, and looking at standard errors (<0.1). As noted on page 8, lines 140-148, the conjoint design of 42 unique patient profiles was created because the design had a D-efficiency of 0.97 (indicating that the subset was representative of the full factorial design) based on all the attributes and levels and because it was possible to minimize standard errors with the given sample size of 400 respondents, which was the target number of respondents. In particular, we determined that a minimum of 8 profiles per respondent were necessary to reduce the standard errors (<0.1) for the conjoint design as a whole and for each attribute as recommended by Orme (*MBC v1.1: Software for Menu-based choice analysis*. 2016. https://sawtoothsoftware.com/uploads/sawtoothsoftware/originals/mbcmanual.pdf).

The low standard errors (<0.1) generated with dummy data from 400 physicians provide strong evidence that our study is adequately powered, as does the fact that the number of profiles generated (3,200) is greater than the sample size of 558 noted above.

According to Bridges et al. (Conjoint Analysis Applications in Health—a Checklist: A Report of the ISPOR Good Research Practices for Conjoint Analysis Task Force. *Value H*e*al*. 2011 Jun;14(4):403–13), *“Sample-size calculations are particularly difficult for conjoint analysis applications in health care. The appropriate sample size depends on the question format, the complexity of the choice tasks, the desired precision of the results, the degree of heterogeneity in the target population, the availability of respondents, and the need to conduct subgroup analyses. Historically, researchers commonly applied rules of thumb, based on the number of attribute levels, to estimate sample size. Orme recommended sample sizes of at least 300 with a minimum of 200 respondents per group for subgroup analysis. Marshall et al. reported that the mean sample size for conjoint analysis studies in health care published between 2005 and 2008 was 259, with nearly 40% of the sample sizes in the range of 100 to 300 respondents.”*

Another concern related to this issue: inside the methodology, the authors state that they designed the survey and profiles based on the 400 respondents. Does it mean that they were aiming to stop the survey at 400 respondents? This needs a clarification.

We have added text on page 12, line 227 to clarify that we had determined that the research would involve 400 respondents prior to starting the research project.

Reviewer #10: To evaluate the impact to physicians of PromarkerD, a biomarker-based blood test predicting the risk of diabetic kidney disease and rapid renal decline, the authors performed a web-based survey asked each physician to make monitoring and treatment decisions about eight randomly selected profiles using a conjoint analysis in 400 physicians. The results indicated that PromarkerD result was most important for increasing the frequency of risk factor monitoring, and second in importance for deciding to prescribe sodium/glucose cotransporter-2 inhibitors (SGLT2s) with a diabetic kidney disease indication, increasing the dose of lisinopril, and replacing ibuprofen with a non-nephrotoxic medication. A high-risk PromarkerD result was associated with significantly higher odds of increasing monitoring frequency, prescribing SGLT2, increasing lisinopril dose, and replacing ibuprofen, whereas a low-risk PromarkerD result was associated with significantly lower odds of increasing monitoring frequency, prescribing SGLT2s, and replacing ibuprofen compared with no PromarkerD results. According to these findings, the authors concluded that PromarkerD could increase adoption of renoprotective interventions in patients at high risk and lower the likelihood of aggressive treatment in those at low risk for renal decline.

The theme of this study is interesting and the manuscript is well written.
I have some comments.

1. How much did the subjects know about PromarkerD? I am asking this because I believe that the subjects’ knowledge of PromarkerD would reflect the results of this study. What are the authors’ thoughts on this point?

**Author response:** Thank you for your detailed review, questions, and suggestions.

We do not believe the respondents had much knowledge about PromarkerD (if any) at the time the study was fielded (in 2020). To ensure that all respondents were basing their responses on the same information about PromarkerD (as opposed to other data or varying conceptions of the test), respondents were presented a single anonymized description of the test. Also, during the phone interviews with five physicians during the survey testing phase, the physicians did not identify the test as PromarkerD after reading the description of the test.

2. It would be interesting to stratify this analysis by primary care physicians and endocrinologists and examine the difference between the two groups. The author described “Separate models for each specialty (PCPs and endocrinologists) were also assessed to identify any significant differences between specialties, but specialty-specific values derived from the conjoint analysis are not included in this manuscript given the increased standard error associated with the smaller sample size for each specialty” (lines 215-218), but it seems possible because the sample size is not small, with approximately 200 subjects in each group.

**Author response:** The manuscript is already quite detailed. We feel that adding the additional results by sub-specialty would over-complicate the findings and detract from the main messaging. In addition, some of the other reviewers believe that even 400 respondents represents a small sample size. Consequently, while we agree a stratification analysis would be interesting, we are refraining from including such an analysis in this manuscript.

3. In accordance with the report of KDIGO Consensus Conference regarding nomenclature for kidney function and disease (Levey AS, et al. Kidney Int 97: 1117-1129, 2020), I propose to change the term “renal” to “kidney”; that is, “rapid renal decline” and “end-stage renal disease” should be changed to “rapid kidney decline” (or “rapid decline in kidney function” would be better) and “end-stage kidney disease”, respectively.

We appreciate you calling our attention to the nomenclature. Because the original survey uses the word “renal” in several places, we will refrain from changing the terminology in this manuscript to avoid confusion. Nevertheless, we will keep your recommendation in mind for future research.

4. The word “ESRD” (line 78) should be spelled out.

You are correct that we should spell out the first mention of ESRD. We have made the change to what is now page 4, line 44.

Reviewer #11: This revised paper was well-written and well responded for reviewer's comments. PromarkerD would be useful to prevent DKD progression. We hope this useful marker could reduce the cost for dialysis for ESRD by diabetes.

Reviewer #12: (No Response)
